# Individualisierte Implantatauswahl bei normal konfigurierter Cochlea: Die Cochleaimplantatchirurgie auf dem Weg zur Präzisionsmedizin

**DOI:** 10.1007/s00106-025-01625-0

**Published:** 2025-04-11

**Authors:** Rolf B. Salcher, Riccardo Di Micco, Max E. Timm

**Affiliations:** 1 Hals-Nasen-Ohrenklinik & DHZ, Medizinische Hochschule Hannover, Hannover, Deutschland; 2 Hearing4all Exzellenzcluster, Hannover, Deutschland; 3 Klinik f. Hals-Nasen-Ohrenheilkunde, Medizinische Hochschule Hannover, Carl-Neuberg-Str. 1, 30625 Hannover, Deutschland

**Keywords:** Cochleaimplantate, Präzisionsmedizin, Genetische Testung, Diagnostische Bildgebung, Minimal-invasive Chirurgie, Cochlear implants, Precision medicine, Genetic testing, Diagnostic Imaging, Minimally invasive surgery

## Abstract

**Hintergrund:**

Aufgrund der großen anatomischen Vielfalt der menschlichen Cochleae existiert keine One-Size-Fits-All-Lösung für die Auswahl des optimalen Elektrodenträgers. Es muss individuell je nach Größe der Cochlea und anderen Faktoren wie dem präoperativ vorhandenen Restgehör entschieden werden.

**Ziele der Arbeit:**

Akzeptanz für Präzisionschirurgie zu schaffen und Grundlagen zu erläutern.

**Material und Methoden:**

Faktoren der Individualisierung werden erläutert und zukünftige Entwicklungen dargestellt.

**Ergebnisse:**

Wenn möglich, stellt die elektroakustische Stimulation die beste Versorgung dar. Bei alleiniger elektrischer Stimulation ist eine Abdeckung der Cochlea von etwa 80 % anzustreben.

**Schlussfolgerungen:**

Die jüngsten Fortschritte in der Cochleaimplantation markieren einen Paradigmenwechsel von standardisierten Lösungen hin zu einer personalisierten Herangehensweise, um somit die Rehabilitationsergebnisse zu optimieren.

## Abstract (en)

**Background:**

Due to the anatomical variety of the human cochlea and the many factors that may determine the choice of the cochlear array, there is no one-size-fits-all method for determining the right implant. The cochlear size, preoperative residual hearing, anatomical variants, operative technique, and cause of deafness must be taken into consideration when making this decision.

**Objective:**

This work aims to spread awareness of precision surgery and provide an overview.

**Materials and techniques:**

Factors that contribute to individualization are explained, and future advances are outlined.

**Results:**

The optimum treatment, if possible, is electroacoustic stimulation. Cochlear coverage of approximately 80% should be the goal when using electrical stimulation alone.

**Conclusion:**

In order to maximize rehabilitation results, recent developments in cochlear implantation mark a paradigm shift away from standardized treatments and toward individualized therapy.

Der Begriff „Individualisierung“ hat in den letzten Jahren zunehmend an Bedeutung gewonnen, insbesondere im Kontext der medizinischen Versorgung. In der Cochleaimplantation bezieht sich Individualisierung auf die Anpassung von Behandlungsansätzen an die spezifischen anatomischen und pathophysiologischen Eigenschaften jedes einzelnen Patienten. Diese maßgeschneiderte Herangehensweise ist entscheidend, um optimale Ergebnisse bei der Hörrehabilitation zu erzielen.

Die anatomische Vielfalt der menschlichen Cochlea spielt eine zentrale Rolle bei der Auswahl des Implantats und der Festlegung der Elektrodenlänge. Fortschritte in der klinischen Bildgebung, insbesondere durch moderne Technologien, ermöglichen eine präzisere Analyse dieser anatomischen Parameter. Diese erlauben eine präzise Bestimmung der cochleären Anatomie und individuelle Frequenzbänder sowie Elektrodenpositionen zu planen.

Darüber hinaus haben objektive Messungen wie eCAP („evoked compound action potential“), Impedanz und Feldtelemetrie (IFT) an Relevanz gewonnen, da sie eine präzise Überprüfung der Funktion und Platzierung des Implantats während des Eingriffs ermöglichen. Diese Technologien tragen dazu bei, die Programmierung des Cochleaimplantats (CI) auf die einzigartigen Merkmale jedes Patienten abzustimmen, was die Sprachwahrnehmung erheblich verbessern kann.

In diesem Referat werden wir die verschiedenen Aspekte der Individualisierung bei der Cochleaimplantation näher beleuchten, einschließlich der Bedeutung anatomischer Variabilität, moderner Bildgebungstechniken und objektiver Messmethoden. Zudem werden wir den individualisierten Ansatz zur Präzisionschirurgie in der CI-Chirurgie vorstellen und die Herausforderungen sowie zukünftigen Entwicklungen in diesem Bereich diskutieren.

## Individualisierung

### Allgemeine Aspekte

Der Begriff „Individualisierung“ entstammt der Soziologie und bezieht sich auf den gesellschaftlichen Wandel, bei dem das Individuum zunehmend in den Mittelpunkt tritt und sich von traditionellen sozialen Bindungen und Normen löst.

### Medizinische Bedeutung

In der modernen Medizin besteht in der Behandlung von Patienten ein Balanceakt zwischen den beiden Ansätzen der individuellen medizinischen Therapie und der einheitlichen medizinischen Therapie. Während die individuelle Therapie auf die spezifischen Merkmale einzelner Patienten eingeht, basiert die einheitliche Therapie auf standardisierten Behandlungsprotokollen, die für große Patientengruppen entwickelt wurden.

In der individuellen medizinischen Therapie werden fortschrittliche Technologien wie genetische Tests, Biomarkeranalysen und patientenspezifische Daten genutzt, um maßgeschneiderte Therapien zu entwickeln. In der einheitlichen medizinischen Therapie basieren die Behandlungen auf Standardprotokollen, die für die Mehrheit der Patienten mit einer bestimmten Krankheit geeignet sind. Diese Therapieform folgt einem „One-Size-Fits-All-Ansatz“ und nutzt standardisierte Diagnosen und Behandlungspläne, die sich in klinischen Studien als wirksam erwiesen haben.

Die Vorteile der individuellen medizinischen Therapie sind eine gezielte Wirksamkeit, reduzierte Nebenwirkungen, frühzeitige Prävention und Anpassung an seltenen Erkrankungen. Demgegenüber stehen höhere Kosten für die personalisierte Diagnostik und Therapie und eine höhere Komplexität, die spezialisierte Fachkenntnisse und Technologien erfordert. Daher variiert die Verfügbarkeit dieser Therapien in Kliniken stark und kann so zu einem ungleichen Zugang für Patienten führen.

Die Vorteile der einheitlichen medizinischen Therapie liegen in einer hohen Kosteneffizienz dank standardisierter Behandlungsprotokolle, einer einfachen Implementierung, schneller Behandlung und gleichberechtigtem Zugang durch die breite Verfügbarkeit standardisierter Behandlungen. Nachteilig bei der einheitlichen medizinischen Therapie sind die begrenzte Individualisierung, da der „One-Size-Fits-All-Ansatz“ nicht immer optimal ist, ein höheres Risiko für Nebenwirkungen birgt, weniger Prävention und unzureichende Lösungen für seltene Erkrankungen beinhaltet.

Die individuelle medizinische Therapie bietet deutliche Vorteile in Bezug auf Wirksamkeit und Personalisierung, ist jedoch teurer und komplexer umzusetzen. Die einheitliche medizinische Therapie ist dagegen kosteneffizienter und einfacher anzuwenden, kann aber in manchen Fällen weniger effektiv sein. Die Bundesärztekammer befürwortet in ihrer Stellungnahme vom 17.01.2020 die Präzisionsmedizin. Sie betont dabei, sowohl die Chancen und Risiken als auch Nutzen und Kosten in Einklang bringen zu wollen.

## Cochleaimplantat

### Indikationsstellung

Cochleaimplantate haben sich als der Goldstandard in der Hörrehabilitation für hochgradig schwerhörige Patienten etabliert. Voraussetzung für den Einsatz eines CI ist das Vorhandensein eines funktionierenden Innenohrs mit intaktem Hörnerv, um die auditorischen Bahnen bis zum Kortex zu stimulieren. Die mehrkanaligen Reizelektroden werden in die Hörschnecke eingeführt, wo sie den Hörnerv elektrisch stimulieren. Da die durch elektrische Stimulation erreichbaren Hörfähigkeiten nicht das Niveau des natürlichen Hörvermögens erreichen, wird ein CI erst dann indiziert, wenn alle anderen Optionen der Hörrehabilitation, wie Hörgeräte oder akustische Implantate, ausgeschöpft sind.

Laut den Leitlinien der Arbeitsgemeinschaft der Wissenschaftlichen Medizinischen Fachgesellschaften e. V. (AWMF) besteht die Indikation für ein CI bei Erwachsenen mit beidseitiger postlingualer Schwerhörigkeit, wenn das Einsilberverstehen unter optimaler Versorgung bei 65 dB weniger als 60 % beträgt. Auch bei einseitiger Taubheit oder Hochtonverlust über 80 dB bei Frequenzen über 1 kHz und gutem Restgehör im Tieftonbereich kann ein CI indiziert werden. Dieses Verfahren wird i. Allg. als elektroakustische Stimulation (EAS) bezeichnet. Die EAS zielt darauf ab, Restgehör mit elektrischer Stimulation zu kombinieren, um das Hören im Hoch- und Mitteltonbereich zu verbessern. Hierbei ist es wichtig, dass das Restgehör während der Elektrodeninsertion zu erhalten, was durch schonende Techniken und den Einsatz kurzer Elektroden erreicht werden kann. Diese Indikationen verdeutlichen die Notwendigkeit einer präzisen Evaluation und Individualisierung des Behandlungsansatzes, um die bestmöglichen Ergebnisse für jeden Patienten zu erzielen.

Viele Faktoren können den Erfolg der Hörrehabilitation bei CI-Nutzern beeinflussen, einschließlich der Erhaltung des Resthörvermögens. Die Analyse der Daten von 808 CI-Empfängern ergab, dass eine längere Taubheitsdauer die CI-Ergebnisse signifikant negativ beeinflusste, wie Blamey et al. 1996 [1] feststellten. Weitere Studien [2–5] identifizierten konsequent auch die Dauer der Taubheit als einen primären Faktor, der die Ergebnisse von Cochleaimplantaten beeinflusst, wobei längere Ertaubungsdauer mit schlechterer Sprachverständnisleistung korreliert. Diese Beziehung wurde in verschiedenen Altersgruppen und Studienentwürfen beobachtet, was ihre Bedeutung für die Vorhersage des Erfolgs von Cochleaimplantaten verstärkt.

Zusätzlich wurden ein höheres Alter bei der Implantation und ein späterer Beginn der Taubheit mit einer schlechteren Sprachwahrnehmung in Verbindung gebracht, insbesondere bei Patienten über 60 Jahren [6]. Kognitive Faktoren, obwohl nicht umfassend untersucht, wurden als potenziell einflussreich festgestellt. Alterungsbedingte Veränderungen der zentralen Verarbeitung könnten ebenfalls die Spracherkennung beeinflussen [7]. Ätiologie war ein weiterer signifikanter Faktor; Patienten mit Meningitis hatten eine geringere Sprachwahrnehmung, während Patienten mit M. Menière im Vergleich zu anderen Ätiologien besser abschnitten.

### Möglichkeiten der Individualisierung

Verschiedene bildgebende Verfahren haben gezeigt, dass die Position des Elektrodenarrays innerhalb der Cochlea die Ergebnisse von Cochleaimplantaten erheblich beeinflusst. Aschendorff et al. [8] stellten fest, dass Arrays, die in der Scala tympani (ST) positioniert sind, zum besseren Sprachverständnis führten im Vergleich zu denen in der Scala vestibuli (SV), wobei bei bestimmten Elektrodenarten vermehrt Skalensprünge beobachtet wurden. Skinner et al. [9] und Finley et al. [10] bestätigten ferner eine negative Korrelation zwischen Elektroden in der SV und der Sprachwahrnehmung, wobei Finley et al. [10] die Elektrodenposition als einen wichtigen Faktor zur Erklärung der Varianz in den Ergebnissen identifizierten. Wanna et al. [11] fanden jedoch keinen Zusammenhang zwischen der Elektrodenposition und dem Sprachverständnis, was die laufenden Debatten über den Einfluss der Elektrodenplatzierung auf die CI-Leistung hervorhebt.

Studien, die die Beziehung zwischen der Tiefe der Elektrodeninsertion bei Cochleaimplantaten und der Sprachverständlichkeit untersuchten, haben gemischte Ergebnisse geliefert. Während einige Forscher [12, 13] positive Korrelationen zwischen tieferen Insertionen und besserer Sprachwahrnehmung fanden, berichteten andere [14, 15] von keiner signifikanten Beziehung. Jedoch wurde auch eine negative Korrelation zwischen der Einführungstiefe und dem Sprachverstehen beobachtet und veröffentlicht. Diese widersprüchlichen Ergebnisse deuten darauf hin, dass der Einfluss der Einführungstiefe auf die Ergebnisse von CI komplex ist und von verschiedenen Faktoren beeinflusst werden kann, was eine weitere Untersuchung rechtfertigt.

Objektive Messungen wie das elektrisch evozierte Summenaktionspotenzial („evoked compound action potential“, eCAP), Impedanz, Feldtelemetrie (IFT) und der evozierte Stapediusreflex-Schwellenwert (eSRT) spielen eine entscheidende Rolle bei der Individualisierung der Cochleaimplantation, indem sie eine genaue Implantatfunktion und -platzierung sicherstellen. Diese Messungen ermöglichen eine maßgeschneiderte Programmierung des CI, basierend auf den einzigartigen anatomischen Merkmalen der Cochlea jedes Patienten, was die audiologischen Ergebnisse erheblich verbessern kann. Außerdem wurde kürzlich intraoperativ eine impedanzbasierte Methode zur Überwachung der Einführung des Elektrodenarrays während einer Cochleaimplantation evaluiert [16].

Aus den genannten Faktoren, die das Sprachverstehen und den Erhalt des Restgehörs beeinflussen können, lassen sich verschiedene Aspekte ableiten, die im Rahmen der individualisierten Cochleaimplantation zur Auswahl des am besten geeigneten Stimulationsverfahrens (reine elektrische Stimulation oder kombinierte elektroakustische Stimulation) und Elektrodenträgers (Länge und Form) vor der Implantation berücksichtigt werden sollten: cochleäre Geometrie, Insertionswinkel und cochleäre Coverage.

Das Weißbuch [17] und die S2k-Leitlinie zur CI-Versorgung [18] legen großen Wert auf eine individualisierte Versorgung, die sich an den spezifischen Bedürfnissen jedes einzelnen Patienten orientiert. Die wichtigsten Aspekte sind:

#### Struktur- und Prozessqualität

Die Versorgung muss in spezialisierten CI-Zentren erfolgen, die über interdisziplinäre Teams (HNO-Ärzte, Audiologen, Logopäden, Psychologen) und adäquate technische Ausstattung verfügen. Qualitätsmanagementsysteme sollen sicherstellen, dass alle Schritte – von der Diagnostik bis zur Nachsorge – standardisiert und auf dem neuesten Stand der Wissenschaft sind. Eine koordinierte Prozesssteuerung durch die CI-versorgende Einrichtung ist essenziell, um eine qualitativ hochwertige und patientenzentrierte Versorgung zu gewährleisten.

#### Präoperative Diagnostik

Eine umfassende Diagnostik soll die Eignung für ein CI klären. Dazu gehören audiologische Tests, bildgebende Verfahren (Magnetresonanztomographie, MRT/Computertomographie, CT) und psychosoziale Evaluierungen. Die Indikationsstellung erfolgt individuell und berücksichtigt Faktoren wie Hörverlustgrad, anatomische Gegebenheiten und Lebensstil.

#### Patientenorientierte Beratung

Patienten sollen über die Funktionsweise, Erfolgsaussichten und Risiken des Implantats aufgeklärt werden. Dabei wird auf individuelle Erwartungen eingegangen. Die Zusammenarbeit mit Patientenorganisationen wird gefördert, um Betroffene in den Entscheidungsprozess einzubinden.

#### Technische Individualisierung

Die Auswahl des Implantatsystems richtet sich nach anatomischen Gegebenheiten (z. B. Elektrodenlänge), Höranforderungen und technischen Präferenzen (z. B. Kompatibilität mit Zusatzgeräten). Zudem finden medizinische Aspekte wie die Ursache der Ertaubung ebenfalls eine zentrale Berücksichtigung bei der Auswahl der Systeme.

#### Postoperative Rehabilitation

Die Rehabilitation wird individuell angepasst und umfasst audiologische Anpassungen des Sprachprozessors sowie hör- und sprachtherapeutische Maßnahmen. Bei Kindern liegt der Fokus auf Habilitation und Spracherwerb, während bei Erwachsenen die Wiederherstellung des Sprachverstehens im Vordergrund steht.

#### Langzeitnachsorge

Eine lebenslange Nachsorge ist essenziell, um die Funktionalität des Implantats sicherzustellen und Anpassungen an veränderte Hörbedürfnisse vorzunehmen. Regelmäßige Kontrollen sollen eine nachhaltige Ergebnisqualität gewährleisten.

#### Evidenzbasierte Leitlinien

Die Leitlinie basiert auf systematischen Literaturrecherchen und Konsensusverfahren unter Einbeziehung von Experten aus verschiedenen Fachbereichen sowie Patientenvertretern. Sie beschreibt alle Versorgungsphasen (Diagnostik, Op., Rehabilitation, Nachsorge) detailliert und betont die Wichtigkeit eines integrativen Ansatzes. Deswegen sollten insbesondere Aspekte der Individualisierung immer kritisch vor dem Hintergrund dieser Leitlinien betrachtet werden.

All die genannten Aspekte des Weißbuchs und der Leitlinie fördern eine qualitativ hochwertige und patientenzentrierte Versorgung mit Cochleaimplantaten. Eine Übersicht über mögliche Faktoren zur Individualisierung und Präzisionsmedizin im Rahmen der CI-Versorgung sind im Anhang in der Tab. 1 dargestellt. Im Folgenden soll v. a. auf die anatomiebasierten Ansätze der CI-Versorgung detaillierter eingegangen werden sowie derzeitige Aspekte der Innovation in dem Gebiet der Hörrehabilitation Erwähnung finden.

**Tab. 1:** Faktoren zur Individualisierung und Präzisionsmedizin im Rahmen der Cochleaimplantat(CI)-Versorgung

Cochleaspezifisch	Operationsspezifisch	Varia/patientenspezifisch
Beginnende Labyrinthfibrose	Gleichzeitige Labyrinthektomie	Genetische Erkrankungen
Labyrinthitis ossificans	Insertionswinkel	Alter
Fehlbildungen	Restgehörerhaltende Technik	Technische Präferenzen
Innenohrschwannome	Fazialis-Mitstimulation	Konnektivität
Cochlealänge	Robotische Insertion	–
–	Elektroakustische Stimulation	–

### Praktische Aspekte

#### Cochleäre Geometrie

**Abb. 1:**
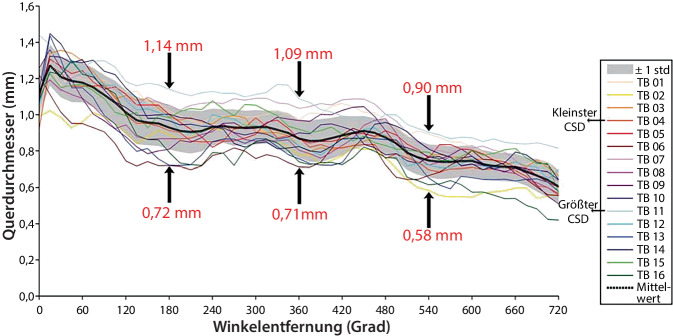
Durchschnittlicher Querdurchmesser (aCSD; *schwarze Kurve*) und individueller Querdurchmesser (CSD; *farbige Kurven*) in Abhängigkeit von der Winkelentfernung, basierend auf dem größten in die Scala tympani im lateralen Bereich für jedes Segment passenden Kreis. Zunahme des aCSD innerhalb der ersten 20° und Abnahme bis zum Ende der zweiten cochleären Windung. *CSD *Querdurchmesser („cross-sectional diameter“), *std.* Standard, *TB* Felsenbeinpräparat („temporal bone”). (Mod. nach Avci et al. [58]; Verwendung gestattet nach Creative Commons Attribution-Non Commercial 4.0 License)

Die Größe und Form der Cochlea variieren erheblich (Abb. 1). Das führt zu unterschiedlichen Insertionswinkeln bei Verwendung desselben Elektrodentyps. Durch Fortschritte in der klinischen Bildgebung in den letzten Jahrzehnten wurde das Wissen über die Vielfalt der menschlichen Anatomie und deren Relevanz für zahlreiche Anwendungen in der klinischen Praxis erheblich erweitert. Durch moderne bildgebende Techniken kann man komplexe dreidimensionale Darstellungen des Innenohrs erstellen, bei denen Knochen, Weichgewebe und neuronale Strukturen differenziert werden können. Auf dieser Grundlage ist es möglich, die anatomische Vielfalt einzelner Innenohrstrukturen noch präziser zu messen.

**Abb. 2:**
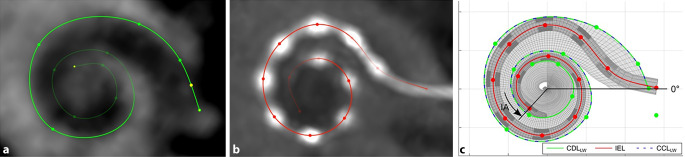
Visualisierung der Methode zur Analyse von Bilddaten. Einsatz präoperativer DVT-Bilder zur Segmentierung der lateralen Wand der Cochlea (**a**). Verwendung postoperativer DVT-Bilder zur Verfolgung des Elektrodenarrays mit Markierung der Position jedes Elektrodenkontakts (**b**). Fusionierung der Bilder zur Rekonstruktion der implantierten Cochlea, zum Mapping verwendbar (**c**). Erläuterung s. Text. *CDL *Gesamtlänge des Ductus cochlearis; *CDLLW *laterale Wand der Cochlea; *DVT *digitale Volumentomographie; *IA* „insertion angle“, Insertionswinkel (Grad); *IEL* „inserted electrode length“, inserierte Elektrodenlänge. (Quelle der Abbildung: Weller et al. [33]; Verwendung gestattet nach Creative Commons Attribution-Non Commercial 4.0 License)

Die Erkenntnisse, die daraus gewonnen werden, sind von zunehmender Bedeutung für die Behandlung von Innenohrschwerhörigkeit mit einem Cochleaimplantat (CI), da sich anhand von audiologischen Ergebnissen Zusammenhänge zwischen Insertionstiefe, Restgehörerhalt und Sprachverständnis nach der Cochleaimplantation ableiten lassen (Abb. 2). Zuerst werden dazu präoperative DVT(Digitale Volumentomographie)-Bilder verwendet, um die laterale Wand der Cochlea zu segmentieren (Abb. 2a). Dann werden die postoperativen DVT-Bilder verwendet, um das Elektrodenarray zu verfolgen, indem die Position jedes Elektrodenkontakts markiert wird (Abb. 2b). Schließlich werden die Bilder fusioniert, um eine Rekonstruktion der implantierten Cochlea zu erhalten, die zum Mapping verwendet werden kann (Abb. 2c).

Basierend auf diesen Erkenntnissen können während der präoperativen Planung individuell für jede Patienten mit normaler, flüssigkeitsgefüllter Cochlea der Implantattyp und die Einführtiefe festgelegt werden, um optimale postoperative Ergebnisse zu erzielen. Die genaue Erfassung der individuellen anatomischen Struktur des Innenohrs im Kontext der Vorbereitung für ein CI und die damit verbundene Vorhersage der erwarteten Elektrodenlage mit verschiedenen Implantaten werden immer wichtiger.

Das Sprachverständnis bei CI-Trägern kann erheblich beeinträchtigt sein, wenn die von den Elektroden gelieferten spektralen Informationen nicht mit ihren tonotopischen Positionen innerhalb der Cochlea übereinstimmen. Eine solche Unstimmigkeit kann dazu führen, dass Frequenzen komprimiert oder erweitert werden, was v. a. die Vokalerkennung beeinträchtigt, ein kritischer Aspekt des Sprachverständnisses. Personen mit postlingualer Taubheit, die bereits mit normaler akustischer Wahrnehmung vertraut waren, leiden besonders unter solchen Veränderungen in der Frequenz [19].

Für Mistri et al. [19] sind die entscheidenden Faktoren in der Frequenzortung, die die Vokalerkennung bestimmen, eine Übereinstimmung zwischen Frequenz und Position, insbesondere im apikalen Bereich, die Menge der gelöschten tiefen Frequenzinformationen und die Anzahl der Stimulationskanäle zur Frequenzauflösung. Diese Faktoren verdeutlichen die Bedeutung einer effektiven Stimulation des apikalen Bereichs der Cochlea für eine verbesserte Vokalerkennung.

Jede Patientin und jeder Patient sollte deshalb einen Elektrodenträger mit der individuellen optimalen Länge erhalten. Damit kann gewährleistet werden, dass eine ideale Frequenzzuordnung von der Basis bis zur Spitze der Cochlea erreicht wird. Gleichzeitig muss eine Überinsertion, wie sie mit einer im Verhältnis zur cochleären Anatomie zu langen Elektrode passieren kann, vermieden werden, ebenso wie mit einem zu kurzen Array in einer langen Cochlea. Es gibt 2 Haupttypen von Elektrodenarrays in der Scala tympani: perimodiolare und laterale Wandelektroden. Das laterale Wandelektrodenarray wird direkt unter dem Corti-Organ platziert und sollte im Idealfall in seiner Länge mit der des Corti-Organs übereinstimmen, gemessen vom Rundfenster bis zum Helicotrema, dem apikalen Punkt der Cochlea.

Algorithmen zur präoperativen Analyse der individuellen Anatomie der Cochlea sind zahlreich entwickelt worden, und deren Einfluss auf den Resthörerhalt nach atraumatischer Implantation wurde untersucht. Die Bestimmung der individuellen Anatomie ist nicht nur bei normal konfigurierten Innenohren wichtig, sondern auch für die verschiedenen Formen von Innenohrmissbildungen. Diese erschweren die Anwendung von Messverfahren, die für die normale Cochlea entwickelt wurden. Deswegen wurde ein multiplanares Regressionsmodell entwickelt und angewendet, um mathematische Gleichungen zu formulieren, die in einer Softwareanwendung zur Schätzung der Länge der luminalen lateralen Wand in malformierten Innenohren verwendet werden kann [20].

Neuerungen in diesem Gebiet sind z. B. die Entwicklung von hochauflösenden optischen Systemen zur Navigation der komplexen Anatomie der Cochlea während der Elektrodeninsertion. Das miniaturisierte optische System wurde in herkömmliche Elektrodenarrays und maßgeschneiderte Cochleasonden integriert und ist mit bereits verfügbaren Robotikplattformen kompatibel, um die klinische Translation zu ermöglichen [21].

Diese Algorithmen können eingesetzt werden, um einen besseren Erhalt des Restgehörs zu erreichen und so das Sprachverstehen zu verbessern.

In einer retrospektiven Analyse wurde z. B. an Patienten, die mit den Elektroden eines einzelnen Herstellers versorgt wurden, gezeigt, dass das Cochleavolumen bei denjenigen Patienten größer war, die nach der Implantation einen Erhalt des Restgehörs aufwiesen, im Vergleich zu denen mit postoperativem Hörverlust [22]. Zudem zeigte sich, die Breite der basalen Windung sowie die Länge der Ductus cochlearis ebenfalls einen Einfluss auf den Erhalt des Restgehörs haben.

Eine weitere Studie [23] belegte ebenfalls, dass eine größere und längere Cochlea mit einer besseren Erhaltung des Restgehörs nach konventioneller Cochleaimplantation assoziiert sind. Dies sind eindrückliche Beispiele, wie anhand der Berücksichtigung der individuellen cochleären Anatomie für die Patienten bestmögliche Ergebnisse bei der Cochleaimplantation erzielt werden können.

#### Insertionswinkel und cochleäre Coverage

Der Winkel der Elektrodeninsertion in die Scala tympani ist entscheidend für die Abdeckung der neuronalen Strukturen in der Cochlea. Aktuelle anatomische Studien zeigen, dass sich diese Strukturen weit apikal erstrecken, was die Notwendigkeit einer präzisen Analyse des Insertionswinkels unterstreicht. Studien haben gezeigt, dass tiefere Insertionswinkel (600–630°) zu einer besseren Hörwahrnehmung führen, da sie eine umfassendere Abdeckung der neuronalen Strukturen ermöglichen [29–31].

Variationen in der Größe und Gestalt der Cochlea beeinflussen ebenfalls die Verteilung der Frequenzen entlang der Spirale der Cochlea. Es wurde nachgewiesen, dass in einer kleinen Cochlea ein Ton mit einer Frequenz von 1 kHz bei ungefähr 18,9 mm (gemessen vom runden Fenster) entlang der Basilarmembran wahrgenommen wird, während dies bei einer großen Cochlea erst bei 22,2 mm der Fall ist. Es ist deshalb von Vorteil, Frequenzen auf den Winkel entlang der cochleären Spirale zu beziehen (gemessen vom runden Fenster), da hierdurch ein großer Teil der Größenunterschiede relativiert wird [24]. Ein Winkel von 180° entspricht ungefähr einer Frequenz von 3 kHz, während 360° einer Frequenz von 1 kHz und 540° einer Frequenz von 400 Hz entsprechen.

Ein entscheidender Faktor beim Einsetzen des Elektrodenträgers in die Scala tympani ist der Winkel der Insertion, der von dem Durchtritt am Rundfenster bis zum apikalen Kontakt des Elektrodenträgers gemessen wird. Man nutzt diesen Winkel beispielsweise, um einzuschätzen, wie weit die neuronalen Strukturen in der Cochlea abgedeckt sind. Aktuelle anatomische Studien des Innenohrs haben gezeigt, dass sich diese neuronalen Strukturen sehr weit apikal in das Innenohr erstrecken [25, 26]. Aufgrund der Vielfalt in der Größe und Form der Cochlea kann ein bestimmter Elektrodenträger in verschiedenen Cochleae sehr unterschiedliche Insertionswinkel erreichen. Avallone et al. [27] haben in einer Gruppe von 91 CI-Patienten demonstriert, dass ein gerades Elektrodenarray mit einer Länge von 28 mm (FLEX28, Fa. MED-EL, Innsbruck, Österreich), das entlang der seitlichen Wand der Cochlea eingeführt wird, Insertionswinkel zwischen 445 und 673° erreichen kann. Ähnliche Varianzen in der postoperativen Elektrodenlage konnten auch für andere Arten von Elektrodenarrays aufgezeigt werden [28–30].

Anbieter von Cochleaimplantaten haben eine breite Auswahl an verschiedenen Systemen im Angebot, die sich hinsichtlich des Durchmessers, der Länge, der Anzahl der Kontakte und der gewünschten Position innerhalb der Cochlea unterscheiden. In den Studien von Canfarotta et al. [31], Helbig et al. [32] und Weller et al. [33] wurde dabei ein optimales Sprachverstehen in Ruhe bei Insertionswinkeln von etwa 600–630° gefunden. Die Ursache für den Zusammenhang zwischen verbessertem Hörvermögen und tieferer Insertion liegt dabei höchstwahrscheinlich in der vollständigeren Abdeckung der intracochleären, neuronalen Strukturen, welche sich maßgeblich über die ersten 2 Windungen der Cochlea verteilen. Die Bereiche der Cochlea, die für die tiefen Frequenzen ab 500 Hz zuständig sind, werden durch Stimulation im Winkelbereich von 540° und darüber aktiviert.

Die cochleäre Coverage („cochlear coverage“, CC) ist der Prozentsatz der Länge der Cochlea, der von einer Elektrode abgedeckt wird, und spielt eine wichtige Rolle für die elektrische Stimulation. Eine präoperative Bildgebung ermöglicht die genaue Bestimmung der Cochlealänge und damit die Auswahl der optimalen Elektrode. Daher spielt für Patienten mit einer reinen elektrischen Stimulation (ES) die CC eine besonders wichtige Rolle. Aber auch für Patienten mit EAS ist die CC wesentlich, um noch intakte Bereiche der Cochlea zu schonen und lange für eine akustische Stimulation nutzbar machen zu können. Es gibt verschiedene Methoden, um eine grobe Schätzung zu machen, wie z. B. die Verwendung der A‑ und B‑Durchmesser der basalen Windung. Für präzisere Ergebnisse wird die multiplanare Regression angewendet. Hierbei wird die Cochlea virtuell abgerollt und die Gesamtlänge der luminalen lateralen Wand des Innenohrs gemessen. Man kann die passende Elektrode in Bezug auf die Länge aus dem gesamten verfügbaren Elektrodenangebot wählen. Wichtig ist, dass eine hohe Anzahl von Zellen der Spiralganglienneuronen (SGN) und der verbleibenden Dendriten durch die elektrische Stimulation aktiviert wird. Die Sinneszellen befinden sich hauptsächlich im Rosenthal-Kanal in der unteren und der zweiten Windung der Cochlea, während sich die Nervenfasern in den Bereichen der Basilarmembran befinden, die für das Restgehör verantwortlich sind. Im Gegensatz dazu wird eine zu knappe Elektrode nicht sämtliche vorhandenen SGN und Dendriten anregen. Es wurde festgestellt, dass die CC stark von der Gesamtlänge der Cochlea abhängt, wenn gerade Lateral-Wall-Elektroden unterschiedlicher Länge verwendet werden. Die Länge der Cochlea an der luminalseitigen Außenwand variiert deutlich von 31–46 mm. Wenn man diesen Aspekt berücksichtigt und die erreichten postoperativen Hörergebnisse analysiert, wird deutlich, dass für eine Abdeckung von 75–80 % die höchsten Werte für die Verständlichkeit der Sprache erreicht werden. Eine niedrigere Abdeckung führt zu einem signifikant schlechteren Ergebnis, während eine höhere Abdeckung ebenfalls zu einem schlechteren Ergebnis führt. Das Ziel besteht daher darin, die Länge der Elektroden so zu bestimmen, dass eine Coverage von ungefähr 75–80 % erreicht wird.

**Abb. 3:**
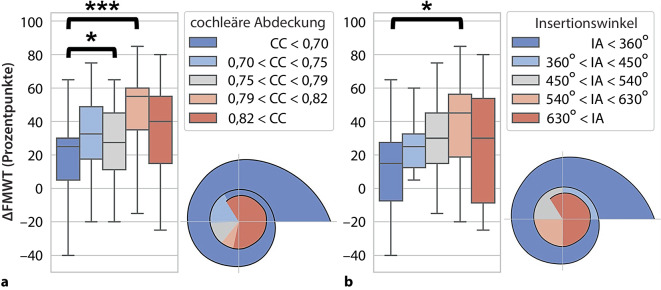
Darstellung der prozentualen Verbesserung im Sprachverstehen des Freiburger Einsilbertests bei 65 dB (*∆FMWT*) für **a **Bereiche unterschiedlicher cochleärer Abdeckung und **b **unterschiedliche Insertionswinkelbereiche (*IA*) für MED-EL-FLEX-Elektroden (Fa. MED-EL, Innsbruck, Österreich). *CC* „cochlear coverage“, cochleäre Abdeckung (%); *FMWT *Freiburg Monosyllabic Word Test, Freiburger Einsilbertest; *IA* „insertion angle“, Insertionswinkel (Grad). (Quelle der Abbildung: Weller et al. [33]; Verwendung gestattet nach Creative Commons Attribution-Non Commercial 4.0 License)

Bei Weller et al. [33] wird sehr schön der Zusammenhang zwischen Insertionswinkel und CC und dem Einfluss auf das Sprachverstehen gezeigt (Abb. 3). Eine CC unter 70 % führte zu signifikant schlechteren Ergebnissen als eine CC von 79–82 %. Die beste Worterkennung zeigte sich bei Insertionswinkeln über 450°, wobei dies noch verbessert werden konnte bei Insertionswinkeln zwischen 540 und 630°.

**Abb. 4:**
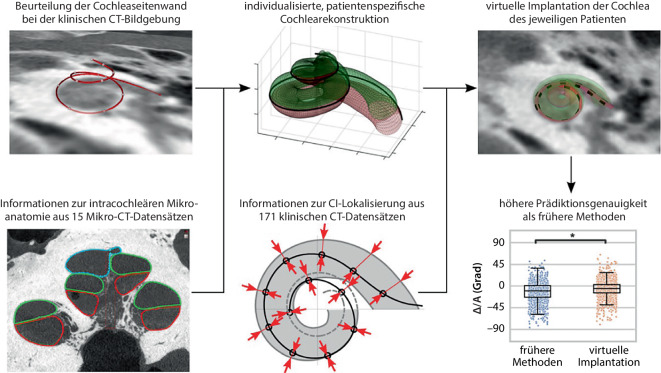
Modell zur dreidimensionalen virtuellen Implantation in die spezifische cochleäre Anatomie eines Patienten mit verschiedenen Elektrodenarraytypen auf der Grundlage der Bildgebungsdatensätze der Computertomographien (*CT*) von 186 menschlichen Cochleae. Modelltraining mit 15 hochauflösenden Mikro-CT-Datensätzen von Leichen-Innenohren sowie anhand von 171 klinischen CT-Datensätzen tatsächlicher Patienten mit Cochleaimplantat (*CI*). Erläuterung s. Text. *∆IA* Unterschied beim Insertionswinkel („insertion angle“). (Mod. nach Schurzig et al. [59]; Verwendung gestattet nach Creative Commons Attribution-Non Commercial 4.0 License)

All diese Entwicklungen zur Erfassung der individuellen cochleären Anatomie, Berücksichtigung der Insertionswinkel und der CC führten zur Entwicklung von softwarebasierten Planungssystemen für die Cochleaimplantation (Abb. 4). Tatsächlich wurde z. B. OTOPLAN® (Fa. CASCINATION, Bern, Schweiz) basierend auf den Fortschritten in der klinischen Bildgebung der letzten Jahrzehnte entwickelt. OTOPLAN® ist ein CE-zertifiziertes Softwaretool, das auf Basis dieser cochleären Größenparameter eine präzise chirurgische Vorplanung ermöglicht. Das Tool ermöglicht es, individuell für jeden Patienten den Implantattyp und die Insertionstiefe festzulegen, um optimale postoperative Ergebnisse zu erzielen. Es berücksichtigt dabei die Zusammenhänge zwischen Insertionstiefe, Restgehörerhalt und Sprachverständnis nach der Implantation, die aus audiologischen Ergebnissen abgeleitet wurden.

Wie in Abb. 4 dargestellt, ermöglichen die aktuellen präoperativen Planungsverfahren bei der CI-Implantation, die Insertionstiefe zu schätzen. Dies ist wichtig, um das Restgehör der Patienten zu schonen und somit das Sprachverstehen zu optimieren. Das vorgestellte Modell ermöglicht eine vollständige, dreidimensionale virtuelle Implantation in die spezifische cochleäre Anatomie eines Patienten mit verschiedenen Elektrodenarraytypen. Um auch die in der klinischen Bildgebung nicht sichtbaren verfeinerten intracochleären Strukturen zu rekonstruieren, wurde das Modell auf der Grundlage von Bildgebungsdatensätzen von 186 menschlichen Cochleae trainiert. Diese bestanden aus 171 Datensätzen von klinischen Computertomographien (CT) tatsächlicher CI-Patienten sowie 15 hochauflösenden Mikro-CT-Datensätzen von Leichen-Innenohren. Die Validierung des Modells erfolgte anhand eines unabhängigen Datensatzes von 141 präoperativen und postoperativen klinischen CT-Aufnahmen von CI-Empfängern.

OTOPLAN ist somit ein Beispiel für die interdisziplinäre Zusammenarbeit zwischen medizinischem, audiologischem und technischem Personal, die darauf abzielt, die Versorgung von hörgeschädigten Patienten durch Präzisionschirurgie in der CI-Chirurgie zu verbessern. Eine Literaturübersicht [34] identifizierte 32 Studien zum klinischen Einsatz von OTOPLAN®, wobei die meisten Studien aus Deutschland stammen. In 22 Studien wurde OTOPLAN® zur Beurteilung der Größe der Cochlea verwendet, gefolgt von der Visualisierung der Elektrodenposition und der dreidimensionalen Segmentierung von Felsenbeinstrukturen. OTOPLAN® ist derzeit der einzige DICOM-Viewer (Digital Imaging and Communications in Medicine) im CI-Bereich, der prä-, intra- und postoperative Bilder verarbeiten kann.

#### Elektroakustische Stimulation

Die elektroakustische Stimulation (EAS) ist ein wichtiger Aspekt der individualisierten Cochleaimplantation, die darauf abzielt, das verbleibende Restgehör optimal mit dem elektrischen Hören zu kombinieren. Durch die eingesetzte Elektrode wird v. a. im Hoch- und Mitteltonbereich das Hören wiederhergestellt, gleichzeitig aber muss bei der Insertion das Restgehör erhalten bleiben, um eine elektroakustische Stimulation durchführen zu können. Die Hybridsysteme stellen die erforderliche technische Grundlage bereit, indem der Sprachprozessor auch ein akustisches Element (Hörgerät) für tiefe Töne enthält. Diese Hörerhaltung ist i. Allg. möglich durch äußerst behutsame Insertion der Elektroden unter Verwendung schonender Operationstechniken mit Live-Monitoring der cochleären Restgehörfunktion während der Insertion. Zusätzlich werden zum Schutz des Innenohrs systemische und/oder lokale Steroide verabreicht. Für diese CI-Versorgung wurden spezielle, kurze Elektrodensysteme konzipiert, die eine maximale Eindringtiefe von 16 mm nicht überschreiten. In der Cochlea erstrecken sie sich etwa 270°, wobei die durchschnittliche Abdeckung bei 44 % liegt. Auf diese Weise können die hohen Frequenzanteile des Hörbereichs funktional wiederhergestellt werden [29, 30]. Es können 3 Kategorien der Hörerhaltung im Tieftonbereich anhand der Veränderung der postoperativen Hörschwellen von 125–1000 Hz identifiziert werden.

- Ein guter Hörerhalt liegt bei weniger als 15 dB Verlust.
- Ein moderater Hörerhalt liegt bei einem Verlust zwischen 15 und 30 dB.
- Ein vollständiger funktionaler Hörverlust tritt bei Verlust von mehr als 30 dB auf.

Mit dieser Kategorisierung ergeben sich für die Hörerhaltung bei kurzen Elektroden, dass etwa 55 % der Patienten eine gute Hörerhaltung haben, etwa 38 % zeigen eine moderate Hörerhaltung, und 7 % erleiden eine Ertaubung.

Die Gefahr einer Schädigung des Gehörs nimmt signifikant zu, wenn längere Elektroden verwendet werden, ebenso wie bei vorgeformten Elektroden [31, 32].

Eine mögliche Erklärung dafür liegt u. a. in der Anatomie der Cochlea. Die Höhe der Scala tympani nimmt von der Basis zur Spitze hin ab, wenn die Einführtiefe etwa 18 mm erreicht. Dadurch steigt die Möglichkeit eines mechanischen Kontakts des Elektrodenträgers mit der Basilarmembran über dieser Einführtiefe, was bei einer sehr flachen Scala tympani zu einer Diskrepanz zwischen dem Durchmesser des Elektrodenkontakts und der Höhe der Scala tympani führen kann. Die Position dieser sog. Hochrisikozone wird u. a. durch die Gesamtlänge und Höhe der Cochlea bestimmt und kann nur begrenzt mithilfe der derzeit verfügbaren klinischen Bildgebungsverfahren festgestellt werden. Wenn die Eindringtiefe mehr als 18 mm beträgt, steigt das Risiko i. Allg. an, und bei der Verwendung von noch längeren Elektroden besteht eine hohe Wahrscheinlichkeit für eine erhebliche Schädigung der Strukturen im Innenohr. Um den Hörerhalt zu verbessern, ist es ratsam, Elektroden zu verwenden, die atraumatisch sind und eine variable Eindringtiefe haben [34].

Die Verwendung von kurzen Elektroden wird kritisch betrachtet, da sie spezifische Einschränkungen und Herausforderungen mit sich bringen. Eine begrenzte Frequenzabdeckung ist aufgrund der kurzen Länge der Elektroden gegeben, die oft nur die erste Windung der Cochlea (etwa 360–450°) erreichen und primär mittlere bis hohe Frequenzen (ab etwa 800 Hz) stimulieren. Tiefe Frequenzen, die essenziell für Klangfülle und natürliche Stimmwahrnehmung sind, können nicht direkt angesprochen werden, wenn das natürliche Restgehör abnimmt oder im Rahmen der Operation verloren geht. Die fehlende Abdeckung tiefer Frequenzen führt zu einer Frequenzverschiebung, die den Klang unnatürlich und blechern wirken lässt. Eine Einschränkung bei der Klangqualität und dem Sprachverstehen kann insbesondere in Situationen mit Hintergrundgeräuschen erlebt werden, wenn tiefe Frequenzen langsam verloren gehen [35, 36].

#### Konzept der partiellen Insertion

Das Konzept der partiellen Insertion spielt eine wichtige Rolle in der Individualisierung der Cochleaimplantation, insbesondere bei Patienten mit Restgehör. Dieser Ansatz berücksichtigt nicht nur die spezifischen anatomischen und audiologischen Gegebenheiten jedes einzelnen Patienten zum Zeitpunkt der Implantation, sondern auch eine mögliche Progredienz des Hörverlusts.

Tatsächlich kann es nicht nur während der Cochleaimplantation zu einem teilweisen oder kompletten Verlust des Restgehörs kommen, sondern auch im Verlauf des Lebens eine natürliche Progredienz der Hörstörung auftreten. In beiden Fällen haben Patienten mit einer kürzeren Elektrode und damit verbunden geringerer CC insbesondere bei Progression des Hörverlusts deutlich schlechtere Hörergebnisse als Patienten mit einer längeren Elektrode und größerer CC. Es gibt also ein Dilemma bei der Entscheidung zwischen guter Hörerhaltung und ausreichender Abdeckung des CI. Wenn ein Patient mit einer kurzen Elektrode nach einer Operation einen Hörverlust erleidet, muss dann eine erneute Implantation mit einer längeren Elektrode erfolgen, um das optimale Hörerlebnis mit einem elektronischen System zu gewährleisten. Deshalb entstand das Konzept der partiellen Insertion. Um die Wahrscheinlichkeit der Hörerhaltung während der Implantation zu maximieren, wird eine lange Elektrode nur teilweise in die Cochlea eingeführt wird, wobei die Länge der Elektrodenträger entsprechend 75–80 % der Gesamtlänge des Innenohrs ausgewählt wird, um bei einer vollen Insertion eine optimale CC erzielen zu können. Es werden bewusst Elektroden außerhalb der Cochlea belassen, um zunächst nur den Bereich elektrisch zu versorgen, der hochgradig schwerhörig ist. Die Elektrode wird in diesem Fall zunächst virtuell so in die Cochlea eingeführt, dass die Spitze der Elektrode sich an dem Ort befindet, an dem die Transitionsfrequenz für den Übergangsbereich zwischen elektrischem und akustischem Hören liegt. Falls der Hörverlust sich über weitere höhere Frequenzen ausweitet, besteht die Möglichkeit, die Elektrode in einem erneuten Eingriff tiefer in die Cochlea einzuführen, was als „Afterloading“ [37] bezeichnet wird. Durch die initial passende Auswahl der Elektrode wird bei vollständiger Einführung eine ausreichende Abdeckung des Hörnervs erreicht, was zu optimaler Hörleistung durch elektrische Stimulation führt.

Obwohl die Verwendung eines individualisierten Elektrodenarrays, basierend auf der Länge der Cochlea, vielversprechend ist, kann dieses auch Nachteile haben. Das Risiko, das Restgehör intraoperativ oder postoperativ trotz Verwendung einer individuell ausgewählten, partiell inserierten Elektrode zu verlieren, sollte nicht unterschätzt werden. Die Notwendigkeit des Afterloadings mit einer erneuten Operation und den damit verbundenen medizinischen, anästhesiologischen und technischen Risiken müssen daher klar mit den Patienten im Voraus besprochen werden.

Die Resultate für partielle Insertion und EAS weisen äußerst positive und überdurchschnittliche Hörergebnisse auf, v. a. beim Verstehen von Sprache im Störgeräusch. Dies zeigt deutlich, wie wichtig das akustische Restgehör für das Verstehen von Sprache ist, besonders in komplexen Hörsituationen. Um die Frequenzen im Mittel- und Hochtonbereich bei einem Hörverlust von über 70 dB durch eine partielle Insertion mit einem elektroakustischen System (ES) angemessen darzustellen, können folgende Parameter zur Bestimmung der erforderlichen Insertionstiefe herangezogen werden:

- verwendbares Restgehör,
- Gesamtlänge des Innenohrs,
- prognostizierte postoperative Hörschwellen im tiefen Frequenzbereich.

In diesem Prozess wird eine Zuweisung von Frequenzen auf der Basilarmembran entsprechend der sog. Greenwood-Funktion durchgeführt. Die Zuordnung von Schallfrequenzen zu den relativen Positionen der Schwingungsmaxima entlang der Basilarmembran wird als Tonotopie bezeichnet und wurde von Greenwood [38] anhand physiologischer Daten weiter präzise quantifiziert.

Sowohl die äußere Spiralform der Cochlea als auch die intracochleären Strukturen unterliegen starken anatomischen Variationen. Für die Beschreibung von Größe und Form der Cochlea werden deshalb in der Literatur diverse Parameter verwendet, um eine spezifische cochleäre Anatomie besser einordnen zu können. Gemäß einer veröffentlichten Übereinkunft zur Beschreibung der Anatomie des Innenohrs [39] wird die Cochleaspirale zunächst anhand ihres Modiolus und des runden Fensters ausgerichtet. Die gängigsten anatomischen Maße im Cochleakoordinatensystem sind der Durchmesser A an der Basis und die Breite B (erstmals beschrieben von Escudé et al. [40], die Höhe H, die Anzahl der Windungen der Spirale (Θ) und die Gesamtlänge des Ductus cochlearis (CDL). Die Frequenzwahrnehmung kann entweder entlang des Corti-Organs (CDLOC) oder entlang der lateralen Wand der Cochlea (CDLLW) gemessen werden, was in Bezug auf die physiologische Wahrnehmung von Frequenzen relevant ist.

**Abb. 5:**
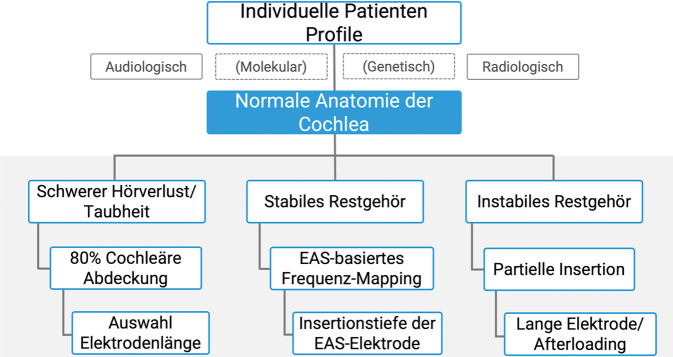
Flussdiagramm zur individuellen Implantatauswahl bei normaler Anatomie der Cochlea. Erläuterung s. Text. Audiologische und radiologische Diagnostik sowie klinischer Verlauf des Hörverlusts als Auswahlkriterien. (Eigentum HNO-Klinik der MHH)

In klinischen Untersuchungen zur Variation der Länge des Innenohrs bezieht man sich üblicherweise auf Messungen der intraluminalen Seitenwand (CDLLW), da diese in CT-Scans von Patienten leicht erkennbar ist. Die anatomischen Unterschiede der menschlichen Cochlea werden auch hier anhand von Bereichen von 30,7–45,0 mm deutlich dargestellt. In der Abb. 5) ist der Entscheidungsprozess zur individuellen Implantatauswahl mit den beeinflussenden Faktoren in einem Flussdiagramm dargestellt: Die audiologische und radiologische Diagnostik bildet aktuell mit dem klinischen Verlauf des Hörverlusts des Patienten die Auswahlkriterien. Molekulare und genetische Diagnostik werden im Rahmen von zukünftigen Biohybridelektroden deutlich an Bedeutung zunehmen.

## Zukünftige Möglichkeiten

### Verbesserungen bestehender CI-Technologie

In den letzten Jahrzehnten gab es keine bahnbrechenden Veränderungen in Bezug auf CI, jedoch können schrittweise Verbesserungen zu einer besseren Sprachwahrnehmung führen. Um den tatsächlichen Nutzen dieser Methoden zu bestimmen, ist eine sorgfältige experimentelle Gestaltung in prospektiven Studien mit Kontrollgruppen und Blindstudien mit hohen Teilnehmerzahlen notwendig. So erhalten wir zuverlässigere Ergebnisse, durch welche technologischen Verfeinerungen sich welche Fortschritte erzielen lassen.

### Neue Wege in der Chirurgie

Es ist grundsätzlich möglich, das Restgehör durch eine schonende Operationsmethode zu erhalten. Zusätzlich kann das sog. cochleäre Monitoring eingesetzt werden. Die Reaktionen der Haarzellen im Innenohr auf akustische Reize, die als cochleäre Mikrofonpotenziale auftreten, ermöglichen nahezu eine Echtzeit-Funktionsüberwachung. Wenn die Elektrode vorgeschoben wird, v. a. über die schon erwähnte 18-mm-Einführtiefe hinaus in die sog. Risikozone, kann es zu einer Verringerung der Amplituden kommen, was darauf hindeutet, dass die cochleäre Mechanik durch die eingeführte Elektrode beeinflusst wird. Wenn die Elektrode weiter vorangetrieben wird, besteht die Gefahr von strukturellen Schäden an der Basilarmembran oder anderen Teilen des Innenohrs, was zu einem signifikanten Hörverlust oder sogar Taubheit führen kann. Wenn die Amplitude abnimmt, kann durch das Stoppen der Einführung, das Zurückziehen der Elektrode oder die Änderung der Einführungsrichtung eine Erholung der Antwortamplitude erreicht werden. So lässt sich die maximale Eindringtiefe für die Erhaltung des Restgehörs bestimmen, um die Hörerhaltung zu maximieren [41–43]. Es besteht eine klare Verbindung zwischen den während der Operation beobachteten Veränderungen und der Hörfähigkeit nach der Operation. Zusätzlich zu den Änderungen der Amplituden werden auch andere Faktoren wie Phasenverschiebungen oder Messungen von mehreren Tönen verwendet, um echte schädigungsrelevante Veränderungen von Amplitudenabnahmen zu unterscheiden, die auftreten, wenn die Aufnahmeelektrode den Generator der frequenzspezifischen Antwort auf der Basilarmembran passiert [43].

Die herkömmliche Operation für Cochleaimplantate ist durch Beschränkungen gekennzeichnet, da ein direkter visueller Einblick in den Einführungsprozess ab dem runden Fenster fehlt. Die Cochlea ist wie eine Black Box. Dies kann durch computer- und roboterassistierte Chirurgie (CAS-RAS) überwunden werden. Menschen unterliegen Einschränkungen ihrer manuellen Fähigkeiten. Sowohl die langsamst mögliche Einführungsgeschwindigkeit als auch Gleichmäßigkeit der Bewegung einer Elektrode ist limitiert. Forschungsergebnisse belegen, dass die Kräfte, die beim Einfügen auftreten, signifikant reduziert werden, wenn die Einführungsgeschwindigkeit unterhalb eines bestimmten Schwellenwerts verlangsamt wird. Dies geht mit einer Verringerung des Risikos für Schäden an den Strukturen des Innenohrs einher.

Um die Genauigkeit der Chirurgie zu verbessern, wurden computer- und robotergestützte chirurgische Verfahren entwickelt oder befinden sich derzeit in der Entwicklung [44–49]. Im Wesentlichen läuft der Prozess wie folgt ab:

Die relevanten anatomischen Strukturen des Felsenbeins werden durch Segmentierung aus einem vor der Operation erstellten CT- oder DVT-Bilddatensatz extrahiert. Die Bestimmung der Einführungstiefe der Elektrode erfolgt unter Berücksichtigung des Restgehörs, das möglichst erhalten bleiben soll. Aus dem Bilddatensatz lässt sich ein Modell der Cochlea erstellen und die Cochleaimplantation virtuell ausführen. Es ist möglich, die anatomisch machbaren Wege für die optimale Platzierung der Elektroden in der Cochlea festzulegen. Mithilfe eines Navigationssystems können sie zum Operationsort übertragen und für das Einsetzen verwendet werden.

Jedoch weisen die ausschließlich auf Navigation basierenden Methoden grundlegende Schwierigkeiten hinsichtlich der erreichbaren Genauigkeit der Planung auf, wenn keine Marker verwendet werden, die am Knochen befestigt sind. Die Verwendung der Trajektorie bei der Platzierung der Elektroden während des Einführens bereitet weiterhin Schwierigkeiten, da die Instrumente nicht präzise genug referenziert und manuell gesteuert werden können.

Deshalb wurden weitere robotische Systeme entwickelt, um die geplante Bahn präzise in einen minimal-invasiven Bohrkanal umzusetzen und die Elektrodeninsertion zu optimieren. Mithilfe passender Bohrsysteme kann ein Bohrkanal von der Oberfläche des Mastoides bis in die Cochlea erstellt werden. Um die geforderte Präzision von weniger als 0,3 mm zu erreichen, kann man auf hochauflösende CT-Verfahren zurückgreifen, knochenverankerte Markersysteme für navigationsgestützte Verfahren einsetzen und starre Fixationssysteme wie die Mayfield-Klemme nutzen, um den Patientensitus mit dem Robotersystem zu verbinden. Auch Bohrschablonen werden verwendet, die speziell angefertigt sind. Der Bohrer und das Einführungswerkzeug können präzise entlang der vorher berechneten Bahn geführt werden. Mithilfe eines Ministereotaxie-Rahmens, der am Kopf der Patienten starr fixiert ist, werden sie in die vorher berechnete Position gebracht.

### Ministereotaxie-Rahmensysteme

**Abb. 6:**
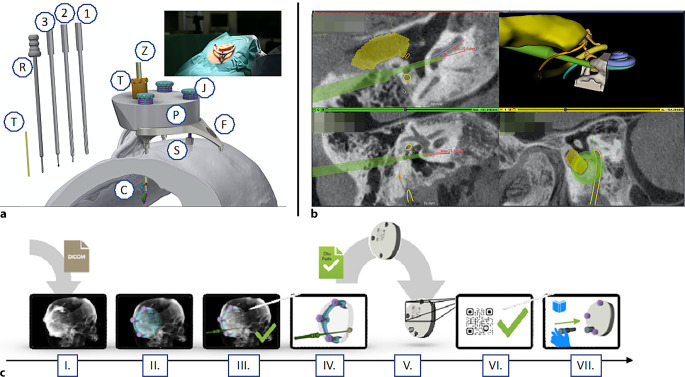
**a** Lage der Cochlea (*C*) in 3–4 cm Tiefe im Felsenbein. Individuell geplanter, gradliniger Zugangsweg (*Z*) bei etwa 80 % der erwachsenen Patienten möglich. Titanrahmen (Mini-Stereotactic Frame, *F*), s. auch *Bild im Bild* mit dem Dummy, während der Op. hinter dem Ohr mit einer Knochenschraube (*S*) befestigt. Minimal-invasive Bohrung mit den OtoDrills 1, 2 und 3 (*1, 2, 3*) und Eröffnung der Cochlea mit dem Round Window Opener (*R*) geführt durch den sog. Tool Guide (*T*) erfolgend. Die Jig Fastener (*J*) zur Befestigung der individuellen Bohrschablone (*P*) dienend. Einführung der CI-Elektrode (nicht abgebildet) durch das Insertionsröhrchen (*I*). (Eigentum HNO Klinik der MHH). **b** Segmentierung der DVT-Bilder mit virtuell 3‑D-geplantem mehrstufigem Bohrpfad. *Gelb* N. VII und äußerer Gehörgang, *Orange* Chorda tympani, *Grün* Bohrpfad, Türkis Scala tympani, *Lila* Scala vestibuli. **c** Workflow: *I* CT-Volumen importieren; *II* Röntgen-Marker lokalisieren; *III* Planung und Kontrolle; *IV* Export und Transfer der Planung; *V* Herstellung der individuellen Bohrschablone; *VI* Verifizierung; *VII* Fixieren der Schablone und mehrstufige Bohrung mit abschließender Insertion (Eigentum HNO Klinik der MHH)

Ministereotaxie-Rahmensysteme wie das OtoJig-Verfahren (Fa. OtoJig GmbH, Hannover, Deutschland) tragen zur Individualisierung der Cochleaimplantation bei, indem dadurch eine präzise präoperative Planung und intraoperative Führung ermöglicht wird. Durch die Verwendung von patientenspezifischen 3‑D-Modellen und Bohrschablonen kann eine optimale Elektrodenpositionierung erreicht werden, was zu einer verbesserten Anpassung an die individuelle Cochleaanatomie führt (Abb. 6). Im Gegensatz zur konventionellen Cochleaimplantation, bei der eine Mastoidektomie und eine posteriore Tympanotomie zwischen N. facialis und der Chorda tympani erfolgt, wird bei der minimal-invasiven Bohrmethode wesentlich weniger Schädelknochen entfernt, um ein CI einzuführen. Außerdem müssen die chirurgischen Landmarken nicht dargestellt werden, und so wird die Gefahr einer ungewollten Verletzung von Strukturen wie z. B. des N. facialis reduziert. Dafür wird eine patientenindividualisierte Bohrschablone genutzt. Diese leitet den Bohrer entlang der geplanten Trajektorie mit Sicherheitsabstand zu allen wichtigen Strukturen auf dem Weg zur Rundfenstermembran der Cochlea. Der Durchmesser der Bohrung kann auf etwa 1,5–3,0 mm reduziert werden. Vorübergehend wird ein steriler Titanrahmen (Mini-Stereotactic Frame) am Schädelknochen während der Operation retroaurikulär angebracht. Auf diesen Rahmen wird zunächst eine Registrierungsschablone montiert, die ausgestattet mit röntgendichten Markern eine automatisierte Registrierung ermöglicht. Zur Überwachung der korrekten Position des Titanrahmens wird während der ganzen Prozedur ein kleiner optischer Referenzmarker an dem Knochen angebracht. Dann wird intraoperativ eine digitale Volumentomographie (DVT) von dem Patienten mit der Registrierungsschablone durchgeführt. Mithilfe einer 3‑D-Planungssoftware (Prototyp, Fa. OtoJig GmbH, Hannover, Deutschland) wird eine dreidimensionale Planung für den besten minimal-invasiven Zugangsweg erstellt. Hierbei werden in einer auf den einzelnen Patienten individualisierten, virtuellen Operation die Sicherheitsabstände zu allen zu schonenden Strukturen festgelegt, wie dem N. facialis, der Chorda tympani, dem Sinus sigmoideus, der Gehörknöchelchenkette und der hinteren Gehörgangswand. Nach dieser 3‑D-Planung wird die Bohrschablone individualisiert für den Patienten angefertigt und in etwa 20 min sterilisiert. Diese individualisierte Positionierungsschablone wird auf dem Titanrahmen befestigt und führt den 3 mm durchmessenden Bohrer (OtoDrill 1) entlang des zuvor definierten Zugangswegs. Anschließend erfolgt die Einführung von 2 kleineren Bohrern (OtoDrill 2 und 3) sowie eines nadelförmigen Öffners (Round Window Opener). Danach werden alle Geräte entfernt, und die CI-Elektrode wird entlang des Bohrkanals durch ein Insertionsröhrchen eingeführt. Das Einführungsröhrchen wird abschließend entfernt und die Elektrode im Kanal fixiert. Die restlichen Schritte erfolgen entsprechend dem klassischen Verfahren.

### Rolle der Genetik

Über 70 % der Kinder, die ein CI zur Behandlung von schwerem Hörverlust oder Taubheit erhalten, leiden an genetischen Ursachen für ihren Hörverlust. Diese Zahl betont die Bedeutung der Genetik für das Verständnis und die Behandlung von Schwerhörigkeiten. Genetische Mutationen können sowohl zu nichtsyndromalen als auch zu syndromalen Formen von Hörverlust führen, wobei nichtsyndromaler sensorineuraler Hörverlust (NSSNHL) am häufigsten vorkommt. Häufig sind einzelne Genmutationen die zugrunde liegende Ursache für einen nichtsyndromalen Hörverlust. Bis zu 20 % der Erwachsenen leiden zusätzlich an genetischen Ursachen für Hörverlust. Durch genetische Tests könnten Ergebnisse vorhergesagt und die Entwicklung personalisierter Behandlungsstrategien nicht nur für Patienten, die sich einer Cochleaimplantation unterziehen, ermöglicht werden. Patienten mit fortschreitendem Hörverlust könnten von individuell zugeschnittenen zukünftigen Behandlungsstrategien profitieren. Die genetischen Ursachen von Hörverlust zu verstehen, kann Kliniker dabei unterstützen, den optimalen Zeitpunkt für Interventionen und die Auswahl des richtigen Geräts zu bestimmen. Es könnte auch die Gesamtwirksamkeit der auditiven Rehabilitation durch die Möglichkeit personalisierter Behandlungsstrategien verbessern.

Durch genetische Tests können spezifische Mutationen identifiziert werden, die mit Hörverlust in Verbindung stehen und aufgrund ihrer begrenzten Pathologie lokalisiert im Innenohr mit besonders herausragenden Ergebnissen der Hörrehabilitation durch ein CI ermöglichen. Zu diesen gehören diejenigen, die die Gene *GJB2*, *SLC26A4* und *MYO15A* beeinflussen.

Die Kenntnis des genetischen Profils jedes einzelnen Patienten ermöglicht eine maßgeschneiderte Festlegung des optimalen Zeitpunkts für die Operation, die optimale Auswahl des CI-Systems und eine bessere prognostische Bewertung hinsichtlich der potenziellen Vorteile der Implantation, was letztendlich die Akzeptanz der Behandlung verbessert.

Kenntnisse über die zugrunde liegende genetische Erkrankung können die klinische Entscheidungsfindung beeinflussen, indem der Fortschritt des Hörverlusts überwacht wird. Auf diese Weise kann der ideale Zeitpunkt für eine Cochleaimplantation bestimmt werden. Durch genetische Analysen kann beispielsweise festgestellt werden, ob die Hörfähigkeit eines Patienten so stark abnimmt, dass sie eine CI-Versorgung rechtfertigt, um dann eine rechtzeitige Intervention sicherzustellen. Darüber hinaus kann die Identifizierung genetischer Faktoren, die mit schlechteren Ergebnissen bei der CI-Versorgung in Verbindung stehen, wie Mutationen, die die Erregbarkeit der Spiralnervenzellen beeinflussen, realistische Erwartungen für die auditive Rehabilitation setzen und entsprechend maßgeschneiderte postoperative Versorgungsstrategien ermöglichen. Durch diesen individuellen Ansatz werden nicht nur die Ergebnisse für jeden Patienten optimiert, sondern es wird auch der Weg für Fortschritte in der Präzisionsmedizin in der Audiologie geebnet.

### Dexamethason freisetzende und Biohybridelektroden

Die Suche nach einer effektiveren Hörrehabilitation mittels Cochleaimplantaten hat zu bedeutenden Fortschritten in der Technologie geführt, insbesondere bei der Entwicklung von sog. Drug-Eluting [53] und biohybriden Elektroden [54, 55], die Medikamente wie Dexamethason freisetzen oder mit autologen mononukleären Zellen beschichtet sind. Diese stellen eine vielversprechende Möglichkeit dar, die Behandlung zu personalisieren und so die Ergebnisse für CI-Träger zu verbessern.

Das CIDEXEL-Projekt („Cochlear Implant DEXamethason ELuting electrode array“) der Fa. MED-EL untersucht die Wirksamkeit und Sicherheit solcher Elektroden. Studien haben gezeigt, dass die lokale Abgabe von Dexamethason das Restgehör besser erhalten und die Elektrodenimpedanzen senken kann [53]. Dies könnte zu einer verbesserten Hörleistung und einem geringeren Energieverbrauch des Implantats führen. Allerdings sind weitere Langzeitstudien erforderlich, um die optimale Dosierung und Freisetzungskinetik zu bestimmen und mögliche Nebenwirkungen auszuschließen.

In der klinischen Studie „Safety and Efficacy of a Drug Eluting Slim Modiolar Electrode Array“ (Clinical Trail NCT06598059) untersucht die Fa. Cochlear Ltd, Sydney, Australien, die CI632D und kann die Effizienz in der Reduktion der Impedanzen als indirekten Marker für Fremdkörperreaktion und Fibroseformation zeigen. Zudem konnte in Tierexperimenten belegt werden, dass diese Art von Elektroden Immunzellen beeinflussen können und darunter insbesondere die Makrophagen, die an der Fremdkörperreaktion und Rekrutierung weiterer Zellen beteiligt sind [56].

Beide Elektrodenträger beinhalten Dexamethason als freisetzenden Strip eingebettet in Silikon auf dem Elektrodenträger und sind Vorreiter für weitere Elektroden, die entwickelt werden können, um lokal Medikamente wie Aracytin oder Wachstumsfaktoren wie „insulin-like growth factor 1“ (IGF1), „hepatocyte growth factor“ (HGF) und Neurotrophin‑3 in die Hörschnecke abzugeben [57]. Die Freisetzung von Dexamethason aus dem Silikon der Elektrodenträger erfolgt relativ schnell und mit begrenzter Kontrolle über die Elutionsdynamik. Offenbar reicht eine frühe Freisetzung von Dexamethason aus, um die akute Entzündungsreaktion zu unterdrücken und langfristig die Fremdkörperreaktion zu hemmen. Eine sehr geringe, kontinuierliche Freisetzung von Dexamethason könnte für die langfristige Unterdrückung der Fremdkörperreaktion genügen bzw. könnte auch die lokale Präsenz von Dexamethason auf der Implantatoberfläche die Proteinadsorption, Komplementaktivierung und Immunzelladhäsion beeinflussen. Diese Erkenntnisse unterstreichen die potenzielle Bedeutung von Dexamethason eluierenden Elektroden für die Verbesserung der Langzeitergebnisse von Cochleaimplantaten [56].

Mehrere klinische Studien (NCT06142682, NCT04750642, NCT06424262) untersuchen derzeit Dexamethason freisetzende Cochleaimplantate mit Fokus auf Sprachverständlichkeit, Hörerhaltung und Elektrodenimpedanzen. Die bemerkenswerte Wirksamkeit dieser Implantate zur Unterdrückung langfristiger intracochleärer Gewebereaktionen auf implantierte Elektrodenarrays, sowohl in Mausmodellen als auch bei menschlichen Probanden, kann für zukünftige klinische Studien und translationale Forschung richtungsgebend sein. Dies könnte letztendlich zu einer Verbesserung der funktionellen Ergebnisse der Cochleaimplantation führen [56].

Die Idee der Biohybridelektroden stellt eine Verschmelzung von biologischen und technologischen Komponenten dar, die darauf abzielt, die Schnittstelle zwischen Cochleaimplantaten und dem umgebenden neuralen Gewebe zu verbessern. Durch den Einsatz von autologen mononukleären Zellen zielen diese Elektroden darauf ab, eine biokompatiblere Umgebung zu schaffen, die eine bessere Integration mit dem auditiven System der Patienten ermöglichen kann. Diese Methode basiert auf der Erkenntnis, dass personalisierte Behandlungsstrategien die Wirksamkeit von CI signifikant steigern können, indem sie individuelle physiologische Unterschiede berücksichtigen.

Die Integration von autologen mononukleären Zellen in das Elektrodendesign erfüllt verschiedene Zwecke. Durch die Verwendung von Zellen der Patienten wird das Risiko von Immunabwehr und Entzündungsreaktionen minimiert, die bei fremden Materialien auftreten können. Die Biokompatibilität ist entscheidend für den langfristigen Erfolg von Implantaten, da sie Komplikationen reduziert, die mit Fibrose und Gewebenarben um die Elektrode herum verbunden sind [54].

Mononukleäre Zellen haben die Fähigkeit, neurotrophe Faktoren auszuschütten, die das Überleben und die Regeneration von Neuronen fördern. Dies ist besonders vorteilhaft im Innenohr, wo die Erhaltung der vorhandenen auditorischen Neuronen für eine optimale Funktion des CI unerlässlich ist. Durch die Verbesserung der neuronalen Unterstützung können diese biohybriden Elektroden die Verarbeitung auditiver Signale und insgesamt die Hörleistung verbessern. Eine aktuelle klinische Untersuchung hat die Langzeitsicherheit dieser Geräte nachgewiesen [55].

Die biohybride Gestaltung eröffnet neue Optionen für lokalisierte Arzneimittelabgabesysteme. Es wäre möglich, die mononukleären Zellen so zu verändern, dass sie spezifische Wachstumsfaktoren oder entzündungshemmende Mittel direkt an der Implantationsstelle freisetzen, um die Heilung und funktionale Integration der Elektroden mit den Geweben des Innenohrs weiter zu fördern.

### Mononukleäre Zellen als antifibrotische und immunmodulatorische Therapien

Mononukleäre Zellen (MNC), insbesondere solche aus autologen Quellen, haben sich als vielversprechende Mittel in der antifibrotischen und immunmodulatorischen Therapie etabliert, v. a. im Zusammenhang mit chronisch entzündlichen Erkrankungen und Gewebereparatur. Ihre vielseitige Rolle bei der Modulation von Immunantworten und der Abschwächung von Fibrose macht sie wertvoll bei der Behandlung von Erkrankungen wie der idiopathischen Lungenfibrose (IPF) und der interstitiellen Lungenerkrankung im Zusammenhang mit systemischer Sklerose.

Die antifibrotischen Mechanismen von MNC beruhen auf ihrer Fähigkeit, verschiedene Zytokine und Wachstumsfaktoren abzusondern, die die Aktivität von Fibroblasten beeinflussen können, was für die Entwicklung von Fibrose entscheidend ist. Beispielsweise können sie das Wachstum von Fibroblasten hemmen und die Ablagerung von extrazellulären Matrixkomponenten reduzieren, wodurch der fibrotische Prozess gemildert wird. Studien haben gezeigt, dass MNC profibrotische Zytokine wie den transformierenden Wachstumsfaktor-beta (TGF-β) und den Tumornekrosefaktor-alpha (TNF-α) herunterregulieren können, die bei der Förderung fibrotischer Veränderungen in Geweben eine zentrale Rolle spielen. Diese antifibrotische Wirkung wurde in verschiedenen experimentellen Modellen beobachtet, was darauf hindeutet, dass MNC zur Wiederherstellung der normalen Gewebestruktur beitragen können, indem sie eine übermäßige Fibrose verhindern.

MNC zeigen neben ihren antifibrotischen Eigenschaften auch signifikante immunmodulatorische Effekte. Durch die Beeinflussung der Produktion von Zytokinen können sie die Immunantwort verändern, indem sie proinflammatorische Wege effektiv dämpfen und gleichzeitig antientzündliche Reaktionen fördern. Diese doppelte Wirkung ist besonders vorteilhaft bei Erkrankungen, die durch chronische Entzündungen gekennzeichnet sind, da sie dazu beiträgt, die Aktivität des Immunsystems auszugleichen und Gewebeschäden zu reduzieren. So haben Makrolidantibiotika, die für ihre immunmodulatorischen Eigenschaften bekannt sind, nachweislich die Funktion von MNC verbessert, indem sie ein günstiges Zytokinmuster fördern, dass die Heilung unterstützt und Entzündungen eindämmt.

Mit dem Fortschreiten der Forschung gehen die potenziellen Anwendungen von biohybriden Elektroden über bloße strukturelle Anpassungen hinaus. Durch die Integration von genetischen Therapien könnte die Behandlung weiter personalisiert werden, indem autologe Zellen modifiziert werden, um ihre regenerativen Fähigkeiten zu verbessern oder spezifische genetische Ursachen von Hörverlust zu behandeln.

Zusätzlich werden laufende Studien untersuchen, wie diese biohybriden Systeme mit fortschrittlichen Signalverarbeitungsalgorithmen in CI interagieren können. Durch die Verknüpfung von technologischen Fortschritten mit biologischen Verbesserungen besteht die Möglichkeit, nächste Generationen von Cochleaimplantaten zu entwickeln, die ein natürlicheres Hörerlebnis bieten.

Die Entwicklung von biohybriden Elektroden, die mit autologen mononukleären Zellen beschichtet sind, stellt einen bedeutenden Schritt in Richtung personalisierter CI-Therapie dar. Durch die Verbesserung der Biokompatibilität, die Unterstützung der neuronalen Gesundheit und die Möglichkeit einer maßgeschneiderten Anpassung bietet dieser innovative Ansatz vielversprechende Aussichten zur Verbesserung der Ergebnisse bei der Wiederherstellung des Hörvermögens. Im Zuge der weiteren Erforschung der Schnittstelle von Biologie und Technologie in der auditiven Rehabilitation nähern wir uns der Verwirklichung einer Zukunft, in der Cochleaimplantate nicht nur wirksam sind, sondern auch individuell auf die einzigartigen Bedürfnisse jedes einzelnen Patienten zugeschnitten sind.

## Ausblick

Für die Zukunft der Cochleaimplantate richtet sich die Aufmerksamkeit auf Fortschritte in der Perilymphanalyse und deren Bedeutung. Die Erforschung der Perilymphe eröffnet neue Perspektiven für das Verständnis und die Verbesserung von CI-Ergebnissen. Moderne molekulare Analysemethoden wie Proteomik, MicroRNA-Profiling und Metabolomik ermöglichen tiefere Einblicke in die biochemischen Prozesse, die den Erfolg einer Hörrehabilitation beeinflussen könnten.

Massenspektrometrische Untersuchungen haben gezeigt, dass spezifische Proteine mit unterschiedlichen Behandlungserfolgen korrelieren. Dabei wurden 5 Proteine bei Patienten mit exzellenten Hörergebnissen häufiger nachgewiesen, während 6 weitere Proteine mit schlechteren Outcomes assoziiert waren [50]. Diese Unterschiede deuten auf komplexe immunologische Mechanismen hin, die den Erfolg eines CI beeinflussen können.

Parallel dazu haben MicroRNA-Analysen weitere molekulare Unterschiede nachgewiesen [51]. Bestimmte miRNA, die an neuronalen Funktionen und Zellüberleben beteiligt sind, zeigen signifikante Expressionsunterschiede zwischen Patienten mit guten und schlechten Hörergebnissen. Diese Erkenntnisse erweitern unser Verständnis der molekularen Grundlagen der Hörrehabilitation.

Metabolische Studien [52] ergänzen diese Perspektive, indem sie die Zusammenhänge zwischen metabolischen Profilen und der Dauer des Hörverlusts untersuchen. Die Analyse kleinster Moleküle und Stoffwechselprodukte könnte Aufschluss darüber geben, wie sich längere Ertaubung auf die biochemische Umgebung des Innenohrs auswirkt.

Diese interdisziplinären Forschungsansätze versprechen eine zunehmend personalisierte Medizin im Bereich der Hörrehabilitation. Zukünftige CI-Versorgungen könnten auf Basis individueller molekularer Profile optimiert werden, was die Erfolgswahrscheinlichkeit und Lebensqualität betroffener Patienten deutlich verbessern könnte.

Die Komplexität der Ergebnisse unterstreicht die Notwendigkeit weiterer Forschung. Große Studien mit umfangreichen Patientenkohorten werden erforderlich sein, um die identifizierten Biomarker zu validieren und ihre prognostische Bedeutung zu präzisieren. Gleichzeitig eröffnen diese Forschungsansätze vielversprechende Perspektiven für ein tieferes Verständnis der Hörrehabilitation.

## Fazit für die Praxis

- Die Fortschritte in der Cochleaimplantation markieren einen Paradigmenwechsel von standardisierten Lösungen hin zu personalisierten Herangehensweisen und optimieren so die Rehabilitationsergebnisse.
- Dabei rücken 3 Kernaspekte in den Fokus:
  - Elektroakustische Stimulation sollte, wo möglich, angestrebt werden.
  - Die Wahl der Elektrode muss auf der spezifischen Anatomie der Cochlea basieren.
  - Elektroden- und Stimulationswahl werden durch individuelle Anamnese und zugrunde liegende Pathologie bestimmt.
- In der Chirurgie stehen folgende Präzisionsaspekte im Mittelpunkt:
  - virtuelle Chirurgie zur optimalen präoperativen Planung der Elektrodenlage,
  - Anwendung atraumatischer Insertionstechniken,
  - kontinuierliches Monitoring des Restgehörs.
- Mit Einführung der Biohybridelektroden öffnet sich das Tor zur Präzisionsmedizin.
- Diese innovativen Ansätze ebnen den Weg für zellbasierte, regenerative Therapien der Schwerhörigkeit und versprechen eine neue Ära in der Behandlung von Hörbeeinträchtigungen.
